# CTX-M β-Lactamase Production and Virulence of *Escherichia coli* K1

**DOI:** 10.3201/eid1512.090928

**Published:** 2009-12

**Authors:** Damien Dubois, Nemani V. Prasadarao, Rahul Mittal, Laurent Bret, Marie Roujou-Gris, Richard Bonnet

**Affiliations:** University of Auvergne, Clermont-Ferrand, France (D. Dubois, R. Bonnet); Teaching Hospital, Clermont-Ferrand (D. Dubois, R. Bonnet); Childrens Hospital, Los Angeles, California, USA (N.V. Prasadarao, R. Mittal); University of Southern California, Los Angeles (N.V. Prasadarao); La Source Hospital, Orléans, France (L. Bret, M. Roujou-Gris)

**Keywords:** Escherichia coli, CTX-M, extended-spectrum β-lactamase, antimicrobial resistance, virulence, meningitis, neonatal, bacteria, dispatch

## Abstract

We report a patient with neonatal meningitis caused by a CTX-M-1–producing *Escherichia coli* K1 strain. The influence of CTX-M production on virulence was investigated in cell culture and a newborn mouse model of meningitis. CTX-M production had no influence on virulence but was a major factor in clinical outcome.

*Escherichia coli* is the second most common cause of neonatal meningitis. Neonatal meningitis *E*. *coli* (NMEC) belong mainly to phylogenetic group B2 and harbor numerous virulence factors (*1*).

Since the beginning of the 21st century, an explosive spread of CTX-M–type extended-spectrum β-lactamases (ESBLs) in *E*. *coli* has occurred (*2*). These enzymes confer resistance to nearly all β-lactam antimicrobial drugs, including third-generation cephalosporins, the first-line treatment for patients with serious *E. coli* infections. However, CTX-M–type ESBLs have been observed mainly in *E*. *coli* strains with few virulence factors or in strains causing minor infections (*3**–**5*). In addition, bacterial resistance to antimicrobial drugs is frequently reported as difficult to reconcile with bacterial virulence (*6*). Highly pathogenic *E*. *coli* such as NMEC are therefore considered susceptible to antimicrobial drugs (*7*). We report a clinical case of neonatal meningitis caused by CTX-M–producing NMEC and the influence of CTX-M production on virulence.

## The Study

In April 2007, a 39-year-old pregnant woman with amniotic sac rupture was admitted to a hospital in Orleans, France, at 28 weeks and 4 days of gestation. Treatment was started with betamethasone (12 mg 1×/d) for fetal lung maturation and amoxicillin (1g 3×/d) for 4 days. Because of a high serum level of C-reactive protein, antimicrobial drug therapy was switched to amoxicillin with clavulanic acid (1g 3×/d) for 1 day. A cesarean delivery was performed at 29 weeks and 2 days of gestation. A lumbar puncture sample of the low-weight (1,560 g) newborn female was tinged with blood. Cerebrospinal fluid (CSF) protein and glucose values were 4.00 g/L and 3.5 mmol/L, respectively. Results of CSF Gram staining were negative.

The infant was admitted to the neonatal critical care unit and received amoxicillin (150 mg), cefotaxime (120 mg), and netilmicin (8 mg) 2×/d for 2 days. Culture of placenta, maternal and infant blood, and infant gastric fluid yielded *E*. *coli*. The isolate was resistant to antimicrobial drugs, including third-generation cephalosporins. Imipenem/cilastatin (25 mg 4×/d) and amikacin (15 mg 2×/d) were given for 2 days. Treatment with imipenem/cilastatin was given for 15 days and then stopped because of the infant’s clinical improvement and return of C-reactive protein to the reference level. Similar drug treatment was administrated to the mother.

One week after drug treatment was discontinued, the infant showed signs of septicemia. A second lumbar puncture sample had protein and glucose levels of 4.56 g/L and 0.1 mmol/L, respectively, and a leukocyte count of 4,700 cells/µL (54% polymorphonuclear cells). *E*. *coli* were isolated from blood and CSF cultures and showed a resistance pattern identical to that of the previous isolate. Meningitis was a complication of the initial sepsis or a relapse of initial unapparent meningitis (*8*).

Treatment was started with imipenem/cilastatin (30 mg 4×/d) for 25 days and amikacin (15 mg 2×/d) for 5 days. Because the infant had a seizure, phenobarbital (22.5 mg) and ciprofloxacin (15 mg 2×/d) were prescribed for 5 additional days. Her condition gradually improved and blood and CSF values returned to reference levels. The infant was discharged from the hospital 1 month later and treatment with the anticonvulsant was discontinued. She showed normal psychomotor development at a regular follow-up pediatric visit.

*E*. *coli* strains isolated from the mother and infant were indistinguishable by enterobacterial repetitive intergenic consensus sequence 2 PCR, random amplified polymorphic DNA analysis, and typing with a MALDI BioTyper (Bruker Daltonique, Wissembourg, France) (*9*). Thus, the isolates corresponded to the same strain, designated Orl-1. PCR-based phylogenetic analysis and serotyping showed that the strain belonged to group B2 and serotype O7:K1:H7, a major O antigen encountered worldwide in NMEC (*1*).

Resistance of the Orl-1 strain (MICs 128 μg/mL for cefotaxime and 8 μg/mL for ceftazidime) was caused by the gene encoding CTX-M-1. This strain was also resistant to tetracycline, trimethoprim, and sulfamethoxazole and susceptible to cefoxitin, imipenem, aminoglycosides, quinolones, chloramphenicol, and fosfomycin. It harbored the major *E*. *coli* genes associated with neonatal meningitis (Table 1) (*1**,**10*).

**Table 1:** Virulence factors of Orl-1 *Escherichia coli* K1 strain, France

Integrative elements* (*1*)	Virulence genes	Present
PAI III_536_–like	*iroN*	Yes
	*sfa/foc*	Yes
GimA-like	*ibeA* (*gimA4*), *ptnC* (*gimA1*)	Yes
PAI II_J96_–like	*hra*	Yes
	*hlyC, cnf1*	No
PAI I_CFT073_–like	*hlyC*	No
	*aer* (*iucC*)	Yes
HPI-like	*fyuA, irp-2*	Yes
GimB-like	*gimB*	Yes
*pks* island†	*clbA, clbK-J, clbP, clbQ*	Yes
Others	*chuA*	Yes
	*ompA*	Yes
	*hek*	Yes
	*iss*	Yes
	*malX*	Yes
	*cdtB-I* to *-V*	No

*PAI, pathogenicity island; HPI, high-pathogenicity island. †Positive cytopathogen effect with transient infection of HeLa cells.

Plasmids from Orl-1 were used to transform *E*. *coli* K-12 DH5α. Three resistance profiles that enabled detection of 3 plasmids were obtained. On the basis of screening of plasmid transformants and Orl-1, most virulence factors genes were presumably chromosomally encoded. Three virulence factors (*aer*, *iss*, and a second copy of *iroN*) were mediated by a tetracycline-resistant, large (≈180 kb), conjugative plasmid (pOrl-1-Te). The 2 other plasmids were pOrl-1-CTX-M-1, the CTX-M-1–encoding large (≈150 kb) conjugative plasmid carrying resistance to trimethoprim and sulfamethoxazole, and pOrl-1-TEM-1, a TEM–1-encoding small (<40 kb) plasmid.

A derivative strain that did not harbor the 3 plasmids (Orl-c) was obtained by plasmid elimination with ethidium bromide. Orl-1, Orl-c, and *E*. *coli* DH5α harboring pOrl-1-CTX-M-1 were tested for invasiveness in human brain microvascular endothelial cells (*10*) and in a newborn mouse (C57BL/6 wild-type) model of meningitis (R. Mittal et al., unpub. data) to investigate the influence of CTX-M-1 production on virulence. *E*. *coli* strain E44, a rifampicin-resistant mutant of archetypical NMEC K1 strain RS218, was used as a positive control (*10*).

Orl-1 and Orl-c strains exhibited 3.5× lower invasiveness than strain E44. However, their ability to invade human brain microvascular endothelial cells was 400× higher than that of strain DH5α–CTX-M1. In the mouse model, DH5α–CTX-M1 did not cause bacteremia or meningitis. In contrast, Orl-1, Orl-c, and E44 induced meningitis (prevalences of 100%, 84%, and 85%, respectively) (Table 2).

**Table 2:** Incidence of meningitis in a newborn mouse model by *Escherichia coli* strain, France*

Bacterial strain	No. animals	Mean ± SD bacteremia, log CFU/mL blood	No. positive CSF cultures (% meningitis)
E44	20	6.95 ± 0.6	17 (85)†
Orl-1	16	6.75 ± 0.8	16 (100)†
Orl-c	17	6.60 ± 0.5	14 (82)†
DH5α-CTX-M1	10	0.10 ± 0.1	0

*CSF, cerebrospinal fluid. †p<0.005, significantly higher than the incidence of meningitis by DH5α-CTX-M1 by χ^2^ test.

The difference between Orl-1 and Orl-c in the mouse model may be explained by loss of plasmid pOrl-1-Te from Orl-c. Plasmid pOrl-1-Te is likely similar to pS88-related plasmids of NMEC because they share 3 virulence factor genes (*iss*, *aer*, and *iroN*) and are large and conjugative. These plasmids contribute to virulence of NMEC (*11*). Orl-1 and Orl-c showed similar behaviors, which suggested that the CTX-M-1–encoding plasmid pOrl-1-CTX-M-1 does not alter virulence of the strain.

Mice with E44- and Orl-1–induced neonatal meningitis were treated with the third-generation cephalosporin cefotaxime, as recommended for humans. Despite antimicrobial drug treatment, Orl-1, but not strain E44, caused meningitis, suggesting that drug resistance is a major factor in clinical outcomes.

## Conclusions

Studies have reported emergence of *E*. *coli* as the predominant organisms responsible for sepsis at any gestational age and for increased rates of drug-resistant *E*. *coli* caused by intrapartum drug prophylaxis (*12*). Spread of ESBLs in *E*. *coli* and intrapartum exposure to antimicrobial drugs may favor emergence of NMEC strains resistant to third-generation cephalosporins.

Two other well-characterized *E*. *coli* K1 strains producing ESBLs have been isolated from patients with neonatal meningitis in Algeria and France. The ESBL was identified as CTX-M-15 in both patients, and 1 infection was lethal (*13**,**14*). Other putative ESBL-producing *E*. *coli* K1 have been recently isolated, especially in developing countries (*15*).

Emergence of ESBL-producing *E*. *coli* strains, which are frequently resistant to fluoroquinolone (*2*), highlights the need for possible alternatives to third-generation cephalosporins for treatment of patients with infected with NMEC. Carbapenems are usually recommended for treatment of infections with ESBL-producing *E*. *coli* (*2*). However, this case report shows the role of treatment duration and the need for additional pharmacokinetic and safety studies in neonates and for adjunctive therapies (*8*).

This characterization of a CTX-M-1–producing NMEC strain highlights the emergence of CTX-M–type ESBL in highly virulent *E*. *coli*. Because of worldwide spread of CTX-Ms, caution should be exercised in the management of patients with NMEC, and first-line treatment for neonatal meningitis may need to be reconsidered.
